# Evaluation of open-face maxillary complete denture for patients with prominent premaxilla: a crossover study

**DOI:** 10.1186/s12903-024-04231-8

**Published:** 2024-04-19

**Authors:** Haitham A. Ismail, Muhammed B. El-Danasory, Salma Abolgheit, Ingy S. Soliman

**Affiliations:** 1 Department of Prosthodontics, Faculty of Dentistry, Alexandria University, Alazarita, Alexandria, Egypt; 2 Department of Prosthodontics, Faculty of Dentistry,, Alexandria University, Alexandria, Egypt; 3 Department of Dental Biomaterials, Faculty of Dentistry, Alexandria University, Alexandria, Egypt; 4 Department of Prosthodontics, Faculty of Dentistry, Alexandria University, Alexandria, Egypt

**Keywords:** Completely edentulous, Esthetics, Open-face maxillary dentures, Prominent premaxilla, Retention

## Abstract

**Background:**

The establishment of good facial esthetics is one of the main objectives of complete denture construction. Unfortunately, it may be the caused issue for patients having a prominent premaxilla due to excessive lip support by the labial flange of the maxillary denture. Open-face dentures (OFD) may suggest suitable prosthetic management for these patients. However, clinical evidence regarding the efficiency of OFD is scarce.

**Methods:**

A total of 38 completely edentulous participants having prominent premaxilla and skeletal class I Angle’s classification were enrolled in this study. Each participant received a mandibular complete denture and 2 opposing maxillary dentures; conventional (CD) and open-face (OFD). On the day of denture insertion, the participants were divided into 2 groups; CD-OFD and OFD-CD where CD-OFD group was instructed to use the mandibular denture and the maxillary CD for 3 months and then to use the maxillary OFD for another 3 months after a wash-out period of 2 weeks. While group OFD-CD was instructed to use the mandibular denture and the maxillary OFD for 3 months then to use the maxillary CD for another 3 months after a wash-out period of 2 weeks. The dislodging force of the maxillary dentures was evaluated using the universal testing machine and the patient perception of retention, esthetics, and comfort was evaluated using the Visual Analogue Scale (VAS). Evaluation was carried out 1 day, 1 month, and 3 months after denture insertion. The Student *t-test* was used to compare the 2 maxillary dentures and the intervals for each denture were compared by using the ANOVA test with repeated measures followed by a Post Hoc test (adjusted Bonferroni) for pairwise comparison.

**Results:**

The significance of the obtained results was judged at the 5% level (*P* value). The dislodging force and patient perception of retention did not show significant differences between the 2 dentures, while the perception of esthetics showed significant differences throughout the follow-up period. Perception of comfort showed an insignificant difference only at the 3-month interval.

**Conclusions:**

Open-face maxillary dentures can be a suitable alternative for patients with prominent premaxilla to achieve satisfactory retention, aesthetics, and comfort.

## Introduction

Complete denture is the routine management for edentulous patients to restore mastication and esthetics [1]. In the era of dental implants, a complete denture is required for implant planning and temporization during the healing period [2]. Furthermore, it is the suitable treatment modality for many clinical scenarios that are not fit for implant placement [3].

Construction of retentive, esthetic, and comfortable dentures requires good supporting residual ridge free of excessive tissue prominences [1]. The presence of a prominent premaxilla interferes with denture placement compromising its retention [4]. Also, the labial flange of the denture increases labial support resulting in unesthetic excessive labial fullness [4]. Preprosthetic alveoloplasty increases morbidity and is not always feasible for the geriatrics. Moreover, it can compromise denture-supporting tissues [5].

The open-face denture can be conservative non-surgical management for patients having prominent premaxilla [6–8]. It can preserve good facial esthetics by eliminating excessive labial fullness [7]. Integrating the use of resilient denture base materials with the design of open-face dentures may allow extending the denture flanges into the undercuts achieving maximum retention while constructing comfortable and esthetic dentures [6–11].

The present study aimed to know the clinical difference between the use of open-face maxillary complete dentures versus conventional ones in the dislodging force and patient satisfaction regarding retention, aesthetics, and comfort. The null hypothesis was that there were no significant differences between the use of the two types of dentures.

## Materials and methods

The present study was conducted as a prospective, double-blinded, randomized controlled clinical trial with a cross-over design. The protocol was approved by the ethical committee of faculty of Dentistry, Alexandria university with ID (IORG 0008839) in 30/04/2023 and retrospectively was first registered on clinical trials with ID number (NCT06209814) on 18/01/2024. Thirty-eight participants indicated for complete dentures were selected from those admitted to the Department of Prosthodontics. Clinical procedures and possible complications were explained to the participants, who signed informed consent for their personal and clinical details along with identifying images to be published in the present study considering the 1975 Declaration of Helsinki, revised in 2013.

**Fig. 1:**
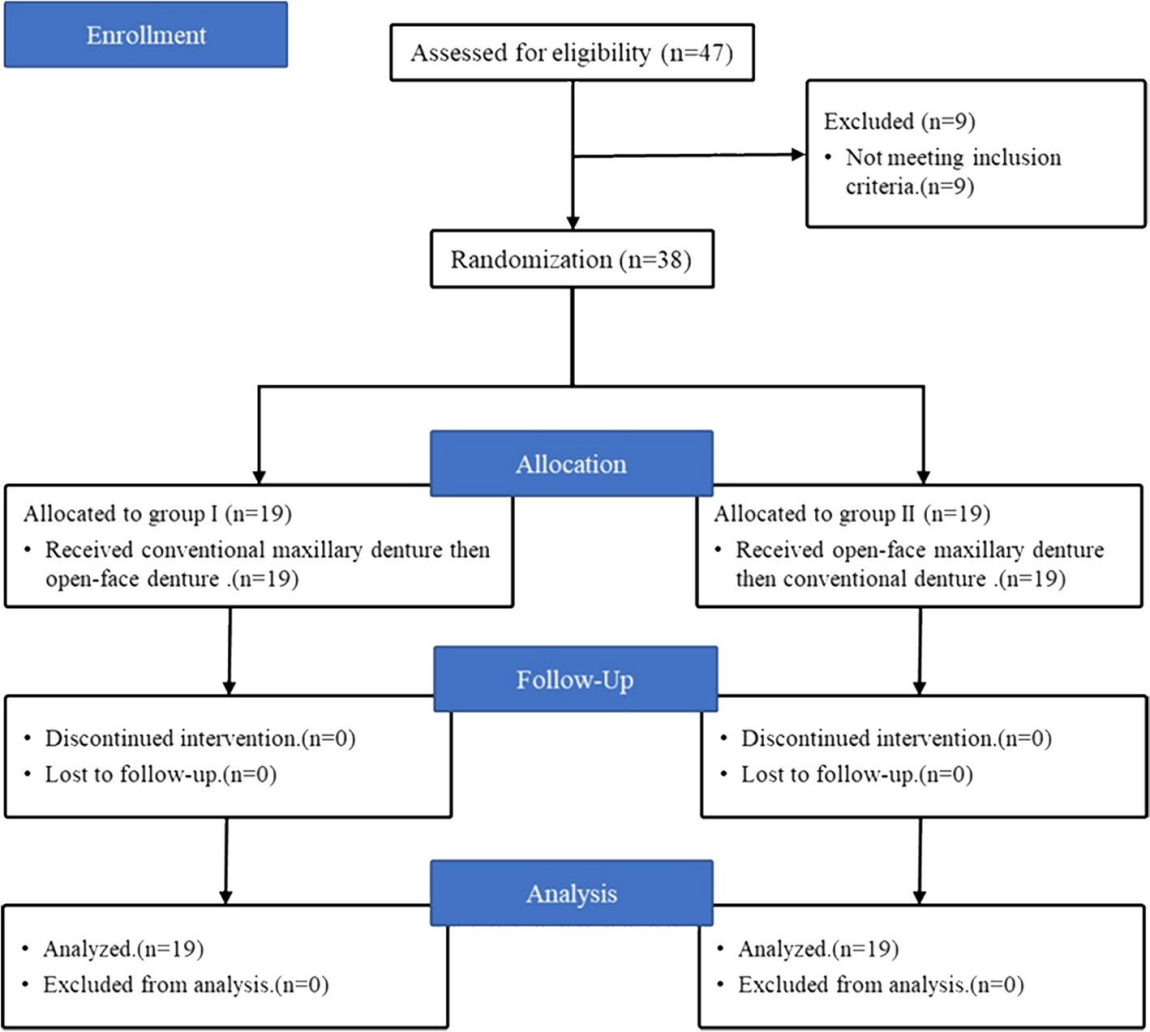
Flowchart diagram of the study

The inclusion criteria were having a completely edentulous ridge with a healthy mucosa and a prominent premaxilla that provided adequate support for the lips, skeletal Class I Angle’s maxillomandibular relationship, and a history of a previous denture with a complaint of unsatisfying esthetics due to over supported lips. Exclusion criteria were xerostomia, presence of tempo-mandibular joint disorder, history of chemotherapy or radiotherapy, uncontrolled diabetes mellitus, Parkinson’s disease, and hypertension. The consolidated Standards of Reporting Trials (CONSORT) 2010 checklist was used to follow appropriate guidelines for the present randomized trial (Fig. 1).

The sample size of 37 participants was calculated by using a software program (G*Power version 3.1.9.2; Heinrich Heine University Düsseldorf) based on the results of a study evaluating the dislodging force between maxillary milled and conventional denture bases [12]. The significance level was set to 95% with a power of 80%. A random allocation sequence was generated by using an online software program (Research Randomizer; http://www.randomizer.org) [13]. Allocation was concealed in opaque envelopes that were opened by the clinician at the appointment of denture insertion. The statistician was blinded to the type of denture being tested. For each participant, a single secondary maxillary impression was poured twice, and 2 identical maxillary record blocks and trial dentures were used at the steps of jaw-relation record and try-in, respectively to ensure the blindness of the clinician till the time of opening the opaque envelope on the day of denture insertion.

Each participant was planned to receive a mandibular conventional denture, a maxillary conventional denture, and a maxillary open-face denture. After a thorough history and diagnosis, preliminary impressions were made using irreversible hydrocolloid impression material (Cavex C37; Cavex) and poured with type III dental stone (Microstone; Whip Mix Corp). Custom trays were made of self-cure acrylic resin (cold cure denture base material; Acrostone) and trimmed to be 2 mm shorter than the vestibular sulcus to allow proper border molding.

**Fig. 2:**
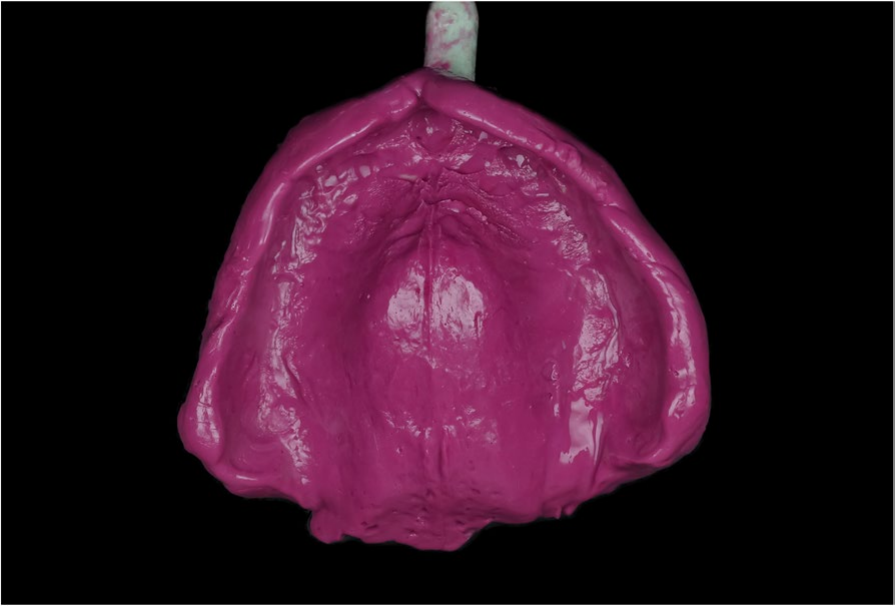
Maxillary polyvinyl siloxane secondary impression

The inner surface of each tray was brushed with a universal tray adhesive (Zhermack, Italy) and left for 15 min for drying then a heavy body polyvinyl siloxane impression material (Aquasil Ultra Heavy regular set; Dentsply Sirona) was used for border molding, and then the definitive impression was made using a medium body polyvinyl siloxane impression material (Aquasil Ultra monophase regular set; Dentsply Sirona) (Fig. 2). The definitive impressions were poured with the type III dental stone. For each patient, the maxillary definitive impression was poured twice to produce 2 definitive casts.

Two identical maxillary record blocks and a mandibular record block were fabricated of the self-cure acrylic resin. The maxillo-mandibular relationship was established for the two maxillary record blocks at the same vertical dimension. A semi-adjustable articulator (Whip Mix 8500; Whip Mix Corp.) was used to establish bilateral balanced occlusion for the 2 maxillary dentures.

**Fig. 3:**
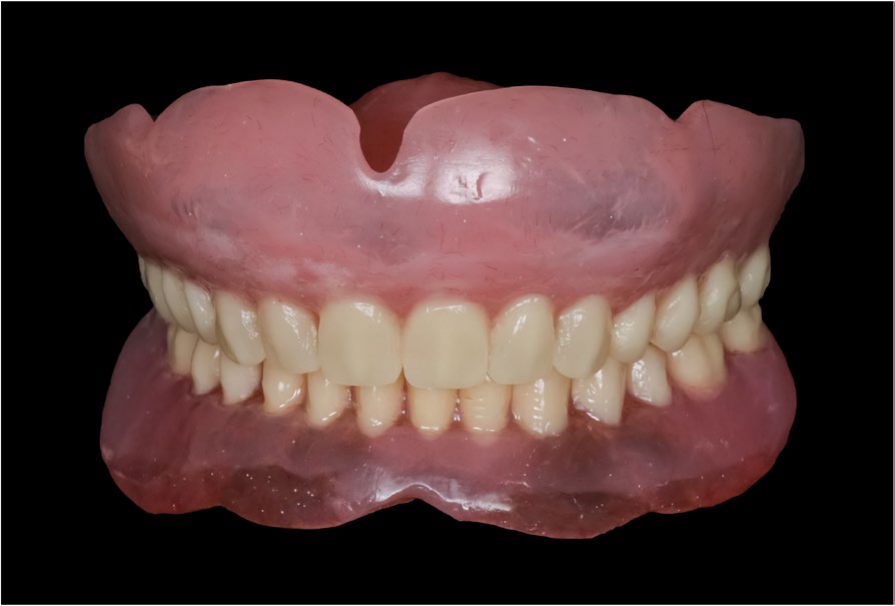
Conventional hard maxillary complete denture with mandibular denture

After clinical try-in and obtaining the patient’s approval, a compression molding technique was used to process the complete dentures. A conventional maxillary complete denture was fabricated of hard heat cure acrylic resin (Vertex SR; Vertex Dental B.V) (Fig. 3).

**Fig. 4:**
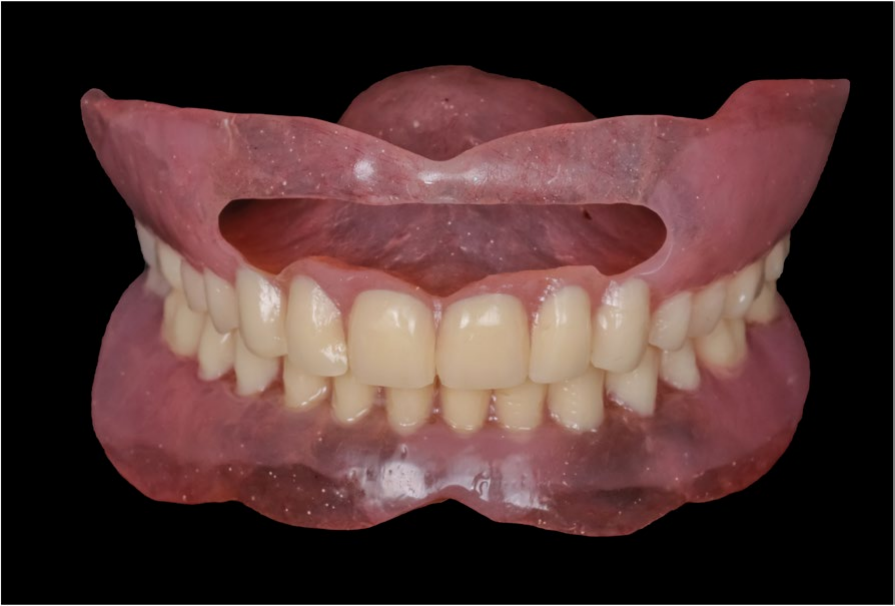
Open-face soft maxillary complete denture with the same mandibular denture

**Fig. 5:**
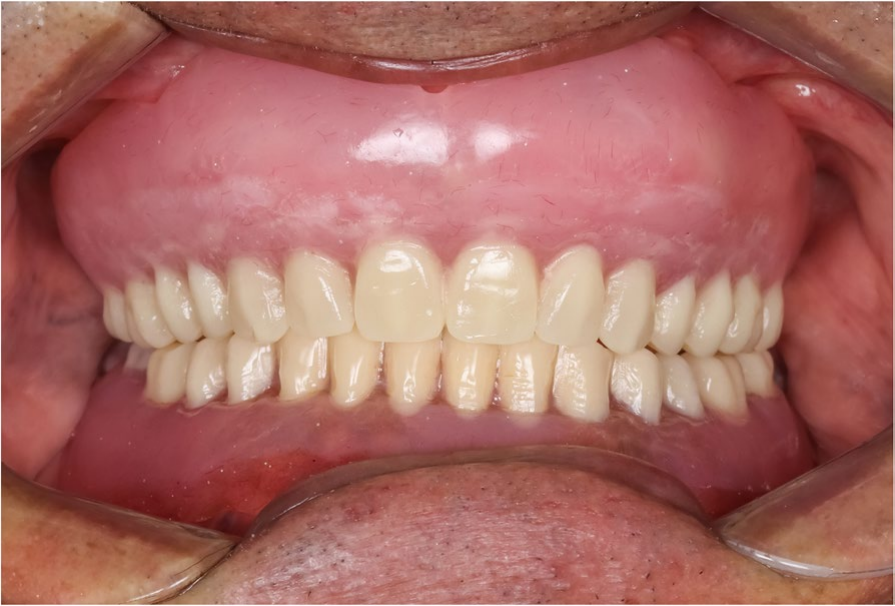
Insertion of the conventional hard maxillary complete denture

**Fig. 6:**
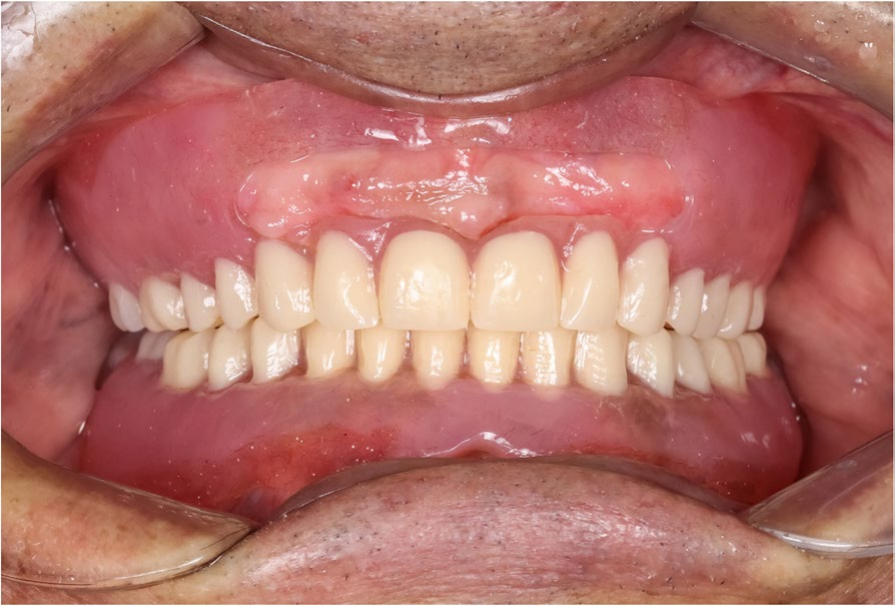
Insertion of the open-face soft maxillary complete denture

For the open-face maxillary denture, the maxillary cast with the trial denture was mounted on a dental surveyor in the zero-tilt position guided by the occlusal plane of the trial denture. The trial denture was removed and the areas of undercut were delineated using the carbon marker. Then the maxillary cast with the trial denture was flasked. At the packing stage, a fold of tin foil was adapted on the cast to act as a spacer then the hard heat cure acrylic resin was packed. After completing the trial closure, the flask was opened and the tin foil was removed and the hard acrylic resin was cut away in the areas apical to the survey line. A resilient denture base material (Vertex Soft; Vertex Dental B.V) was packed and the flask was closed for curing. After deflasking, the dentures were finished and polished. For the open-face maxillary denture, the labial flanges were windowed to uncover the prominent area of the premaxilla (Fig. 4). At the appointment of denture insertion (Figs. 5, 6), the concealment envelope was opened by the clinician to assign the participant to the group CD-OFD or OFD-CD.

Group CD-OFD: Conventional maxillary (CD) and mandibular complete dentures were delivered, and the participant was instructed to use these dentures for 3 months. The open-face denture (OFD) was stored in an incubator in a water bath at 37 ± 1°C [14]. After a washout period of 2 weeks [15, 16], the conventional maxillary denture was replaced by the open-face denture (OFD), and the participant was instructed to use it for 3 months.

Group OFD-CD: Open-face maxillary (OFD) and conventional mandibular complete dentures were delivered, and the participant was instructed to use these dentures for 3 months. The conventional denture (CD) was stored in an incubator in a water bath at 37 ± 1°C [14]. After a washout period of 2 weeks [15, 16], the open-face maxillary denture was replaced by the conventional denture (CD), and the participant was instructed to use it for 3 months.

**Fig. 7:**
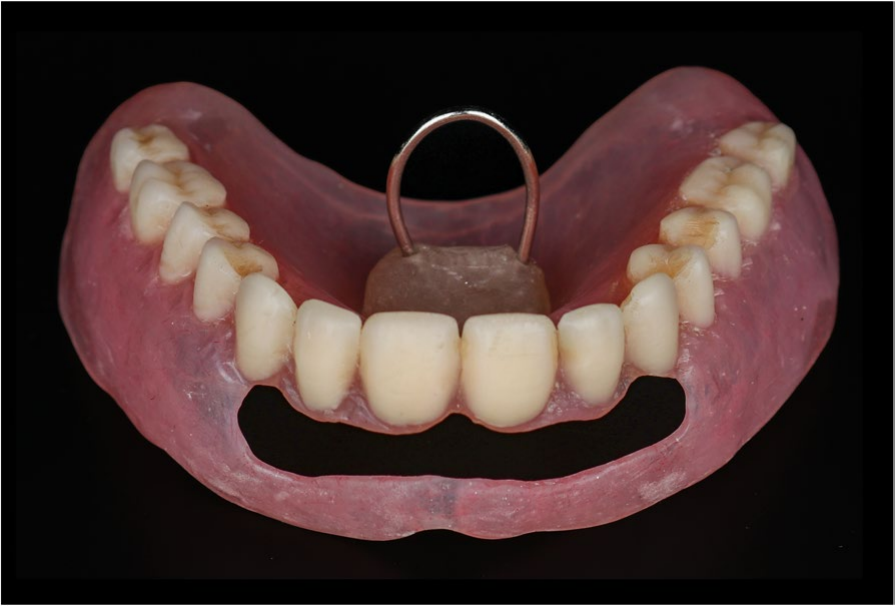
Determination of the geometric center of the maxillary denture

**Fig. 8:**
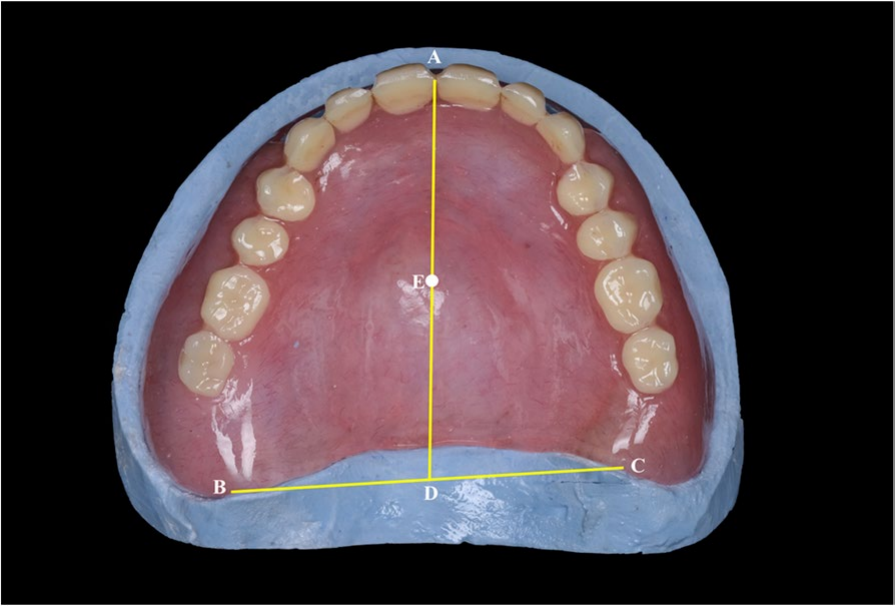
Determination of the geometric center of the maxillary denture

**Fig. 9:**
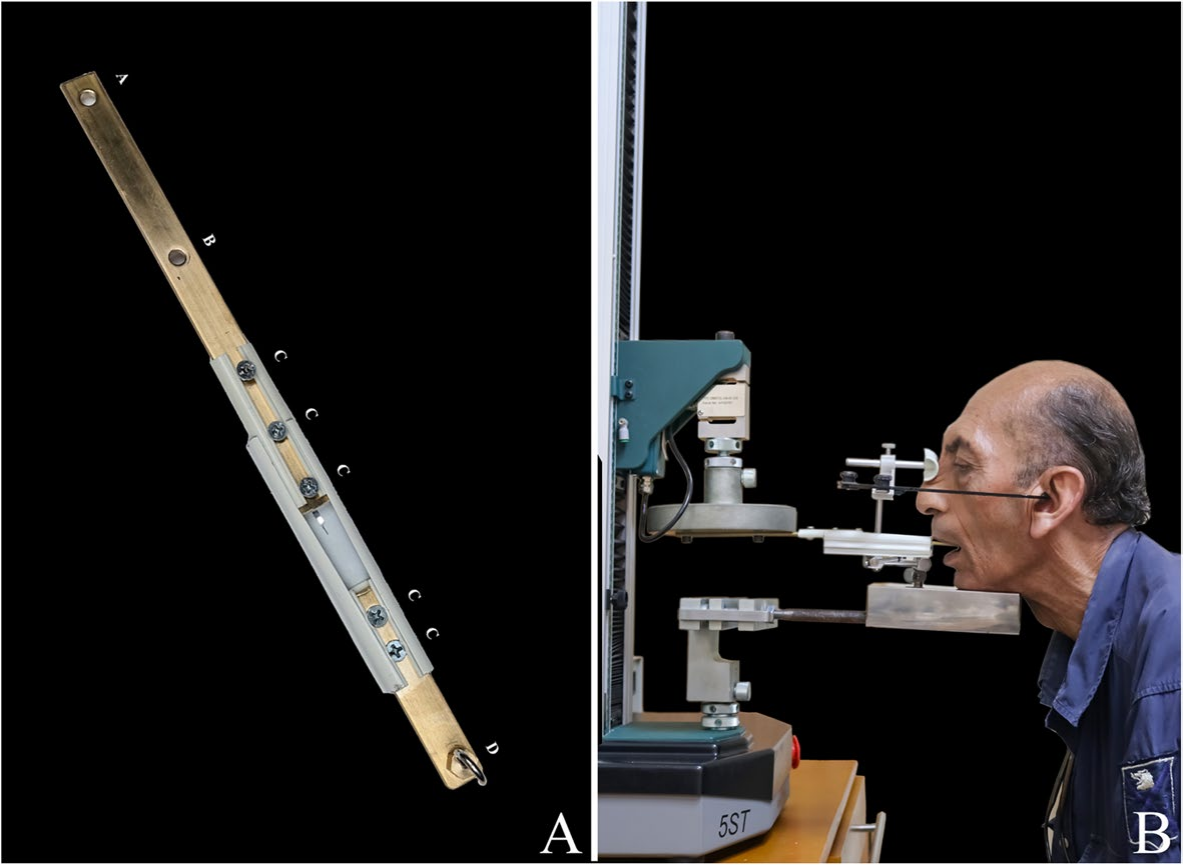
**A** Force transmission device (FTD), **A** and **B**; holes for attachment to the universal testing machine, **C**; nuts for adjusting the length of FTD, **D**; hook for attachment to the denture and **B** Patient positioning for testing the retention of the maxillary denture

The retention of the maxillary dentures and patient satisfaction were evaluated at 1 day (20 min after denture insertion) [17], 1 month, and 3 months after denture insertion. The denture was examined for any complaints before retention evaluation at different intervals. Retention was evaluated by using a universal testing machine (5ST; Tinius Olsen) to exert a vertical dislodging force on the maxillary denture [12]. A stainless steel loop was attached to the geometric center of the maxillary denture by using an autopolymerizing acrylic resin (Fig. 7) [12]. The center of the maxillary denture was determined as follows (Fig. 8) [12]. The center of the labial frenum (point A) and the hamular notches (points B and C) were located in the denture base [12]. The distance halfway between points B and C was measured, and the location was marked on the posterior border of the denture base (point D). Finally, half the distance between points A and D was marked as the center of the denture base (point E) [12]. A force transmission device (FTD) (Fig. 9A) was used to transmit the dislodging vertical force from the universal testing machine to the maxillary denture. The FTD is a split bar with a hook at an end to engage the loop attached to the denture. The bar has nuts for adjusting its length. A facebow was used to set the Frankfort plane of the patient parallel to the floor (Fig. 9B). After orienting the participant to the universal testing machine, it was set to exert a dislodging force at a rate of 50 mm/min [12]. The retention test was repeated 3 times for each denture and the mean value was calculated.

Participant satisfaction was evaluated regarding retention, esthetics, and comfort by using visual analog scales. The level of satisfaction was indicated as a sign on a 10 cm Visual analog scale (VAS) [18], labeled with (not at all satisfied) at the zero end with (completely satisfied) at the other end. The distance (mm) between the zero point and the sign marked by the patient on the 10-cm-line was measured and expressed in percentage. To evaluate the perception of retention, esthetics, and comfort, the participants were respectively asked the following questions; How do you find the retention of the maxillary denture? How much are you satisfied with your facial appearance? and How much are you comfortable with the maxillary denture?

The statistical tests were performed by using a software package (IBM SPSS Statistics, v20.0; IBM Corp). The Shapiro–Wilk test of normality was used to verify the normal distribution of collected data. The dislodging force and the parameters of patient satisfaction were described as mean and standard deviation (SD). The Student *t-test* to compare the 2 maxillary dentures. The three studied intervals for each group were compared by using the ANOVA test with repeated measures followed by a Post Hoc test (adjusted Bonferroni) for pairwise comparison. The significance of the obtained results was judged at the 5% level.

## Results

Thirty-eight participants met the inclusion criteria. The permuted block randomization technique with variable block size was used to allocate the participants to equal groups. Participants included 23 (62.2%) men and 15 (37.8%) women with a mean age of 57 years. The dislodging force of the conventional denture (CD) showed a statistically significant improvement throughout the three intervals (1st day, 1 month, and 3 months) (*P* < 0.001) as well as for the open-face denture (OFD) through the same intervals (*P* < 0.013). Meanwhile, on comparing the (CD) to the (OFD) across the three-time intervals there was a statistically insignificant difference favoring the (CD). (*P* = 0.638, 0.434, and 0.499) respectively (Table 1).

**Table 1:** Mean ± standard deviation of dislodging force (N) and retention evaluation (%) of conventional (CD) and open-face (OFD) maxillary dentures at different intervals

	**Dislodging force**				**Retention**			
	**1st day**	**1st month**	**3rd month**	***P***	**1st day**	**1st month**	**3rd month**	***P***
**CD** **(*n* = 38)**	44.57 ± 12.69	45.70 ± 12.60	46.07 ± 12.39	< 0.001^*^	79.38 ± 8.04	81.08 ± 6.63	82.70 ± 5.49	< 0.001^*^
**OFD** **(*n* = 38)**	43.29 ± 10.58^a^	43.63 ± 9.86^a,b^	44.13 ± 9.42^b^	0.013^*^	80.46 ± 7.08^c^	80.89 ± 7.38^c^	82.14 ± 6.31	< 0.001^*^
***P***	0.638	0.434	0.499		0.541	0.908	0.681	

*The P*-value for Student t-test for comparing between the two groups. *P*-value for Post Hoc Test (adjusted Bonferroni) between periods in each group. Letters indicate insignificant differences, and ^*^ indicates significant differences

For patient perception, denture retention of (CD) and (OFD) showed a statistically significant enhancement throughout the three intervals (1st day, 1 month, and 3 months) (*P* < 0.001 and < 0.001). However, on comparing the (CD) to the (OFD) across the same intervals, there was a statistically insignificant difference (*P* = 0.541, 0.908, and 0.681) respectively (Table 1).

On comparing the aesthetics evaluation of (CD), the results showed a statistically significant improvement throughout the three intervals (*P* < 0.001). On the other hand, the (OFD) results across the same intervals showed a statistically insignificant difference (*P* = 0.162). As for comparing the (CD) to the (OFD) across the same intervals, there was a statistically significant difference supporting the (OFD) (*P* < 0.001, < 0.001, and < 0.001) (Table 2).

**Table 2:** Mean ± standard deviation of esthetics (%) and comfort (%) evaluation of conventional (CD) and open-face (OFD) maxillary dentures at different intervals

	**Esthetics**				**Comfort**			
	**1st day**	**1st month**	**3rd month**	***P***	**1st day**	**1st month**	**3rd month**	***P***
**CD** **(*n* = 38)**	79.59 ± 6.22^a^	80.92 ± 5.59^a^	84.22 ± 5.57	< 0.001^*^	80.27 ± 5.90	83.27 ± 4.93	89.05 ± 3.25	< 0.001^*^
**OFD** **(*n* = 38)**	88.95 ± 3.78	88.92 ± 3.87	89.73 ± 3.65	0.162	83.43 ± 6.08	89.54 ± 4.41^e^	90.19 ± 4.49^e^	< 0.001^*^
***P***	< 0.001^*^	< 0.001^*^	< 0.001^*^		0.026^*^	< 0.001^*^	0.217	

*The P*-value for Student t-test for comparing between the two groups. *P*-value for Post Hoc Test (adjusted Bonferroni) between periods in each group. Letters indicate insignificant differences, and ^*^ indicates significant differences

Whereas, for comfort evaluation of (CD) and (OFD) independently, the results showed a statistically significant progression through the three intervals (*P* < 0.001 and < 0.001) respectively. While comparing the results of the (CD) and (OFD) for the intervals (1 day and 1 month) there were statistically significant differences (*P* = 0.026*,* and < 0.001) respectively favoring the (OFD). While for the interval (3 months) there was a statistically insignificant superiority for the (OFD) (*P* = 0.217) (Table 2).

## Discussion

The null hypothesis regarding the dislodging force and retention was not rejected but rejected regarding patient satisfaction with esthetic and comfort. Good retention is very important for the success of the complete denture, however facial esthetic and comfort are determinant factors [19–21]. Denture esthetics has an initial impact on patient satisfaction as it affects patient’s self-esteem and social acceptance.

While conventional and open-face denture dentures achieved close results regarding retention evaluation, there were significant differences between them regarding esthetics and comfort favoring the open-face dentures.

Close retention results may be owed to the need for making relief at the areas of bony prominences and the borders of the conventional dentures to relieve pressure areas and to allow easy insertion and removal of the denture decreasing their retention scores. Meanwhile, the soft borders of the open-face dentures allowed them to easily engage mucosal undercuts eliminating the need for relief. Also, close fitting of the open-face dentures and completing the soft tissue drape by the upper lip may achieve a peripheral seal compensating for the labial window [22, 23].

Efforts were exerted to standardize the measurement of the dislodging force for each maxillary denture. A loop was attached to the geometrical center of the maxillary denture and the facebow was used to align the Frankfort plane parallel to the floor [12]. Hence, most of the resulting dislodging force exerted by the universal testing machine was, almost, in a vertical direction which may not simulate the directions of the affecting dislodging forces during mastication or speech. Therefore, the dislodging force evaluation was augmented with the evaluation of patient perception of denture retention to assess the efficiency of the used maxillary denture.

Previous Crossover studies comparing between different types of complete dentures applied a washout period of 2–4 weeks [15, 16, 24]. In the current study, a washout period of 2 weeks was found to be sufficient to eliminate the effect of the previous denture as indicated by the presence of healthy mucosa which was free of inflammation, ulcerations, and indentations.

Unlike retention parameters, satisfaction with esthetics and comfort was significantly different between the two types of maxillary dentures except for comfort at the last interval. The different results regarding esthetics may be a result of the advantage of the open-face denture to eliminate excessive lip fullness providing a natural-looking appearance unlike the conventional denture. The use of resilient denture base material for open-face dentures can provide a good explanation for the reported higher comfort. The high initial satisfaction with esthetics and comfort was reflected in the faster adaptation of the patients to the open-face denture. The insignificant difference in patient perception of comfort after 3 months can be explained by continuous post-insertion adjustments and the neuromuscular adaptation developed during the follow-up period.

To our knowledge, there are no available studies evaluating the open-face denture to evaluate the reported results. Hence, more randomized clinical trials are required to establish strong evidence-based practice. The limitations of this study included the small number of patients. The larger the study sample size, the smaller the margin of error and more precise results therefore similar studies with larger sample size is recommended.

## Conclusion

Based on the findings of this clinically controlled crossover study, for cases having prominent premaxilla, it can be concluded that:

1. The Open-face complete denture with resilient flanges has dislodging force and patient perception of retention very similar to those the conventional dentures.
2. The open-face complete denture with resilient flanges can achieve significantly superior results regarding patient perception of esthetics and comfort compared to those achieved by the conventional denture.

## Data Availability

All datasets and materials used and/or analyzed during the current study are included in this manuscript.
